# Small molecule induced polymerization of BCL6 facilitates SIAH1 mediated degradation

**DOI:** 10.1038/s41392-021-00556-w

**Published:** 2021-04-01

**Authors:** Sandeep Rana, Amarnath Natarajan

**Affiliations:** 1 Division of Preclinical Innovation, National Center for Advancing Translational Sciences, National Institutes of Health, Rockville, MD USA; 2 Eppley Institute for Cancer Research, Fred & Pamela Buffett Cancer Center, University of Nebraska Medical Center, Omaha, NE USA

**Keywords:** Chemical biology, Structural biology

The recent report by Slabicki et al. in *Nature* used an array of techniques including cryo-electron microscopy to elucidate the mechanism of action of BI-3802, a molecular glue.^1^

The discovery of small molecules that highjack cellular quality control machinery to selectively degrade proteins has generated considerable excitement in the drug discovery community, particularly toward targets often deemed “undruggable”. Whether its proteolysis targeting chimeras (PROTACs), consisting of heterobifunctional molecules connected by a linker that facilitates the recruitment of an E3 ligase to the protein of interest (POI) or small molecules that function as molecular glues^2^ to induce a novel interaction between an E3 ligase and the POI, the ultimate goal is to tag the POI for destruction in cells. PROTAC design is relatively straight forward, wherein a POI binding small molecule, and an E3 ligase binding ligand are connected through a linker that enables stable ternary complex formation (POI:PROTAC:E3 ligase) followed by POI degradation. On the other hand, the discovery of molecular glues has largely been happy accidents. Approaches for the de novo design of molecular glues are in their infancy and the identification of molecular glues have typically occurred serendipitously and characterized retrospectively.

**Fig. 1:**
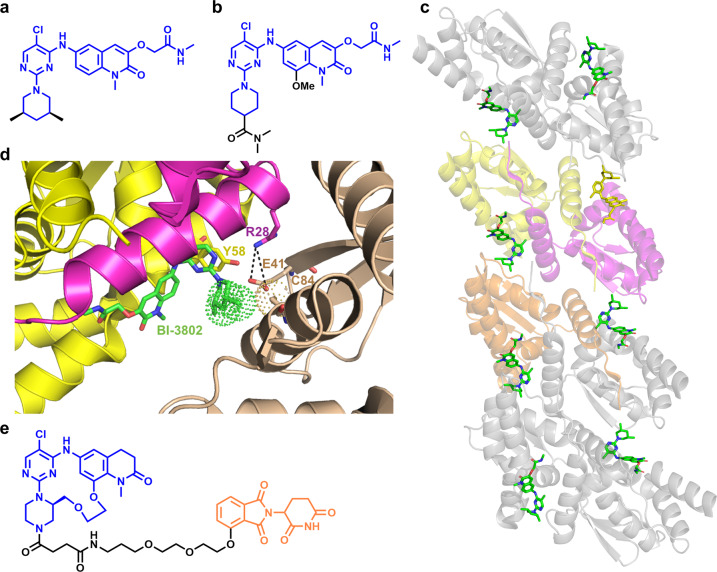
Small molecule BCL6 modulators and the binding mode of the BCL6 molecular glue. **a** Structure of BCL6 molecular glue BI-3802. **b** Structure of BCL6 inhibitor BI-3812. **c** Cyro-EM model of BCL6 filament with BI-3802 PDB code (6XMX). **d** Binding mode and key interaction made by BI-3802 (green) at the interface of BTBα (yellow), BTBβ (magenta), and BTBγ (orange) domains. **e** Structure of BCL6 PROTAC

The transcription factor BCL6 is a known oncogenic driver of lymphoid malignancies such as diffuse large B cell lymphoma (DLBCL). BCL6, through the Broad-Complex, Tramtrack, and Bric a brac (BTB) domain, recruits co-repressor complexes resulting in transcriptional repression. In mice, BCL6 knock out results in a severe inflammatory phenotype, while mutations that disrupt BCL6-corepressor interaction only blocks the formation of germinal centers, the source of mutated B-cells. Kerres et al., at Boehringer Ingelheim performed a high throughput screen to identify inhibitors of BCL6(BTB)-corepressor interaction. Structure–activity relationship studies with the hits followed by biophysical and cellular studies identified compounds, such as BI-3802 that induced BCL6 degradation and inhibitors such as BI-3812 that disrupted the BCL6 co-repressor interaction (Fig. 1a, b).^3^ Although competition studies with MLN4924 suggested that BI-3802-mediated BCL6 degradation was cullin-independent, the exact mechanism remained elusive. Compared to the BI-3812 inhibitor, the degrader BI-3802 robustly de-repressed BCL6 target genes and exhibited anti-proliferative effects.

Slabicki et al. used quantitative mass spectrometry studies to show selective degradation of BCL6 upon BI-3802 treatment, whereas the BCL6 inhibitor BI-3812 treatment did not alter BCL6 levels, which was consistent with the report by Kerres et al. BI-3802 induced non-cullin E3 ligase mediated degradation of BCL6 was elucidated using an elegant fluorescent reporter assay system. Pre-treatment with MLN7423, an inhibitor of ubiquitin-activating enzyme or MLN132, an inhibitor of 26S proteasome, rescued BI-3802 induced BCL6 degradation, whereas no such effect was observed with MLN4924, an inhibitor of neddylation pathway. Truncation studies established that the first 275 amino acids of BCL6 were sufficient for BI-3802 induced BCL6 degradation in cells. Live cell fluorescence microscopy revealed reversible BI-3802 induced foci formation in cells expressing a non-degradable ^eGFP^BCL6 which indicated polymerization.

Negative-stain electron microscopy (EM) showed that incubation of recombinant BCL6 with BI-3802 resulted in the formation of highly ordered helical filaments. Computational modeling using RosettaDock 4.0 revealed favorable energetics to facilitate oligomerization through the symmetrical association of BI-3802 bound BCL6 dimers. The pitch and the radius of helical polymers derived from modeling studies were similar to those observed in the EM study. Cyro-EM density derived from BCL6-BTB homo-octamer was resolved using the crystal structure of BI-3802 bound BCL6 BTB domain (Fig. 1c). BI-3802 occupies the interface between BCL6 homodimers and is stabilized by π–π interactions between Y58 of BCL6 and the pyrimidine ring of BI-3802. The solvent exposed hydrophobic 3,5-dimethylpiperidine fragment in BI-3802 associates with the hydrophobic side chain of C84 of the adjacent dimer to enable higher order assembly. Overlays show that steric hindrance of 4-dimethylacetamide moiety in the BCL6 inhibitor BI-3812 prevents the hydrophobic interaction required for BCL6 oligomerization. The interdimer hydrophobic interaction was further stabilized by an intermolecular interaction between R28 and E41 (Fig. 1d, e), which was confirmed by point mutation studies. The structural studies were complemented by unbiased mutational studies that showed BI-3802 induced BCL6 oligomerization is required for the cellular foci formation and BCL6 degradation, which is responsible for the cellular toxicity. Complementary genome-wide CRISPR–Cas9 genetic screens were used to identify SIAH1 as the non-cullin E3-ligase that ubiquitinates BI-3802 induced BCL6 oligomers. Validation studies also revealed that SIAH1 is the endogenous E3-ligase that facilitates BCL6 degradation. An earlier observation i.e., the loss of degradation with the BCL6 truncation mutant that polymerized, was due to the absence of the SIAH1 substrate-binding motif VxP. Reciprocal immunoprecipitation and in vitro ubiquitination assays with various BCL6 mutants established “SIAH1 as a bonafide E3 ligase of BCL6”.

The work performed by Slabicki et al. elucidated the mechanism of action of a molecular glue BI-3802 induced BCL6 degradation and identified SIAH1 as the BCL6 E3-ligase.^1^ This work, for the first time, showed that a small molecule induced polymerization of a POI followed by its degradation as a viable therapeutic modality. McCoull et al. reported a BCL6 PROTAC by conjugating an optimized macrocyclic BCL6 inhibitor to a phthalimide based E3 ligase ligand (Fig. 1f). The weak anti-proliferative effects observed with the BCL6 PROTAC were attributed to the partial degradation of BCL6 in different subcellular compartments.^4^ In addition to the degradation of BCL6, the ability of the molecular glue BI-3802 to cause mislocalization and inhibition of BCL6 could explain its improved anti-proliferative effects. Moreover, unlike bivalent PROTACs that are often large and have unfavorable ADME properties, molecular glues that induce degradation such as BI-3802, phthalimides and fulvestrant are smaller in size and possess favorable drug like properties.

The study by Slabicki et al. also suggests that careful examination of compound induced in vitro aggregation of proteins could yield novel molecular glues. Novel approaches to identify molecular glues that facilitate protein degradation will offer additional leads for the drug discovery pipeline. Can modifications to the surface exposed functional groups of small molecules mimic point mutations on the protein surfaces that facilitate their supramolecular assemblies is an intriguing question?^5^
